# The oncoprotein H-Ras^V12 ^increases mitochondrial metabolism

**DOI:** 10.1186/1476-4598-6-77

**Published:** 2007-12-01

**Authors:** Sucheta Telang, Andrew N Lane, Kristin K Nelson, Sengodagounder Arumugam, Jason Chesney

**Affiliations:** 1 Molecular Targets Group, Department of Medicine, James Graham Brown Cancer Center, University of Louisville, Louisville, Kentucky 40202, USA; 2 Structural Biology Program, Department of Medicine, James Graham Brown Cancer Center, University of Louisville, Louisville, Kentucky 40202, USA

## Abstract

**Background:**

Neoplastic cells increase glycolysis in order to produce anabolic precursors and energy within the hypoxic environment of a tumor. Ras signaling is activated in several cancers and has been found to regulate metabolism by enhancing glycolytic flux to lactate. We examined the effects of sequential immortalization and H-Ras^V12^-transformation of human bronchial epithelial cells on the anabolic fate of fully-labeled ^13^C-glucose-derived carbons using two-dimensional total correlated spectroscopic analysis-nuclear magnetic resonance spectroscopy (2D TOCSY-NMR).

**Results:**

We found that the introduction of activated H-Ras^V12 ^into immortalized human bronchial epithelial cells unexpectedly increased tricarboxylic acid cycle activity as measured by the direct conversion of ^13^C-glucose carbons into the anabolic substrates glutamate/glutamine, aspartate and uridine. We then observed that immortalization and H-Ras^V12^-transformation of bronchial epithelial cells caused a stepwise increase in oxygen consumption, a global measure of electron transport chain activity. Importantly, ectopic expression of H-Ras^V12 ^sensitized immortalized cells to the ATP-depleting and cytotoxic effects of electron transport perturbation using the complex I inhibitor rotenone.

**Conclusion:**

Taken together, these data indicate that the oncoprotein H-Ras^V12 ^increases mitochondrial metabolism and provide new rationale for the targeting of the tricarboxylic acid cycle and electron transport chain as anti-neoplastic strategies.

## Background

Biosynthesis of proteins, nucleic acids, lipids and complex carbohydrates requires coupling to the hydrolysis of nucleoside triphosphates (ATP and GTP in protein biosynthesis, CTP and UTP in lipid and carbohydrate biosynthesis), as well as the incorporation of carbon and nitrogen from metabolic precursors. Maintaining Na^+^/K^+ ^ion gradients and other ion gradients for transport consumes a large fraction of the ATP generated in resting (G0/G1) cells, and activating macromolecule biosynthesis in preparation for cell division requires the production of additional ATP equivalents. This need can be met under aerobic conditions by increasing the flux of acetyl coenzyme A into the tricarboxylic acid cycle (and via anaplerotic reactions to replenish carbon used for biosynthesis) derived from glucose, amino acid and fatty acid oxidation. However, cancer cells have a tendency to increase the glycolytic flux even under aerobic conditions, and secrete a large fraction of the glucose carbons as lactate [1-7]. This implies a bypass of oxidative phosphorylation, such that glycolysis alone accounts for a substantial fraction of the ATP generation. This enhanced aerobic glycolysis is known as the Warburg effect [8,9]. However, the precise mix of fuels that drive cancer and normal epithelial cells is controversial, and may well be dependent on cell type and the growth conditions [3,10].

The Ras family of GTPases (H-, K- and N-Ras) function to transduce signals from receptor tyrosine kinases (*e.g*. epidermal growth factor receptor) that promote cell survival and proliferation [11]. Activating mutations of the Ras GTPases that cause insensitivity to inhibitory GTPase-activating proteins are among the most common genetic alterations detected in human cancers [11]. Given the central signaling role of Ras in cell proliferation and tumorigenesis, it is not surprising that these oncoproteins also mediate neoplastic metabolism. Ectopic expression of oncogenic Ras in immortalized cells has been found to: (i) increase glucose uptake and lactate production; (ii) increase ribose-5-phosphate, a glucose-derived anabolic precursor of DNA and RNA; and (iii) increase sensitivity to the glycolytic inhibitors, 2-deoxyglucose and oxamate [12-14]. However normal, non-immortalized human bronchial epithelial cells supplemented with growth factors were recently observed to consume glucose and secrete lactate at a similar rate as Ras^V12^-transformed human bronchial epithelial cells [15]. Accordingly, the precise downstream metabolic effects of Ras signaling within the setting of immortalized cells and in comparison to matched primary cells have not been well established.

By monitoring the flow of ^13^C from fully labeled glucose into metabolites, it is possible to determine the major pathways that are influenced by immortalization and transformation, including the activity of glycolysis, the oxidative and non-oxidative pentose phosphate pathways, the tricarboxylic acid cycle and pyrimidine biosynthesis. Herein, we report that the introduction of oncogenic H-Ras^V12 ^into immortalized bronchial epithelial cells increases the conversion of ^13^C from labeled glucose into several shunt products of the tricarboxylic acid cycle. Furthermore, we find that H-Ras^V12 ^increases oxygen consumption and sensitizes the immortalized cells to electron transport chain disruption. Importantly, the observed increase in mitochondrial metabolism caused by H-Ras^V12 ^may prove useful for the development of agents that selectively disrupt the metabolism of neoplastic cells.

## Results

### Glucose consumption and lactate secretion by normal, hT/LT-immortalized and H-Ras^V12^-transformed human bronchial epithelial cells

**Figure 1:**
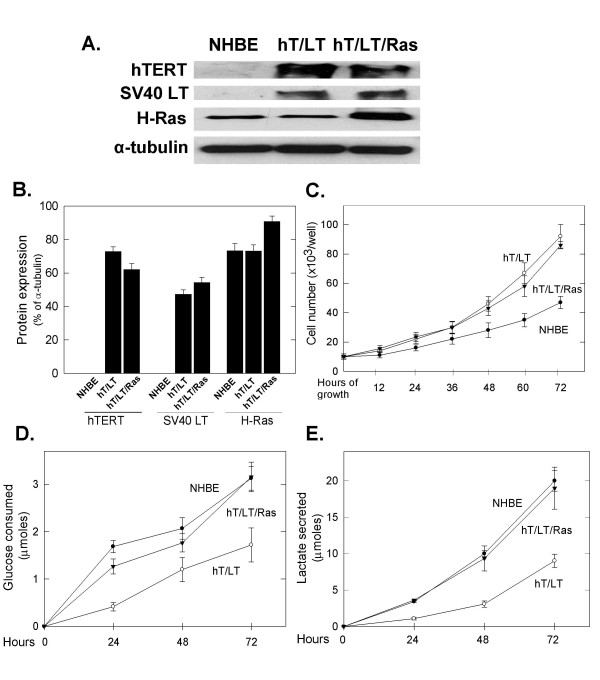
**Proliferation, glucose consumption and lactate secretion by NHBE, hT/LT immortalized and hT/LT/Ras-transformed cells**. a. Protein extracted from normal human bronchial epithelial cells (NHBE), NHBE cells transduced with telomerase and large T antigen (hT/LT) and subsequently with H-ras^V12 ^(hT/LT/Ras) was resolved on a 4–20% gradient gel, transferred to a PVDF membrane and expression of human telomerase (hTERT), SV40 large T antigen (LT), H-Ras and α-tubulin was examined by Western blot analysis. b. Protein expression in NHBE, hT/LT and hT/LT/Ras cells by Western blot was quantified by densitometric analyses and expressed as percentage of control (α-tubulin). c. Primary, immortalized and H-Ras^V12^-transformed bronchial epithelial cells were cultured in growth media and enumerated after the indicated time periods. d. Unconsumed glucose remaining in the media was measured after 24, 48 and 72 hrs expressed as the (calculated) consumed glucose. e. Lactate secreted into the media by the cells was measured after 24, 48 and 72 hrs. d. Data are expressed as the mean ± SD of four experiments (b, c) and five experiments (d, e).

In order to examine the metabolic effects of mutant Ras in cells relevant to human cancer, we obtained normal human bronchial epithelial (NHBE) cells that had been sequentially immortalized and transformed using the telomerase catalytic subunit (hT), SV40 large T antigen (LT) and an oncogenic allele of ras (H-Ras^V12^) [16]. We confirmed that the normal human bronchial epithelial (NHBE) cells had been stably transfected with the telomerase catalytic subunit (hT), SV40 large T antigen (LT) +/- oncogenic H-Ras^V12 ^by Western blot analysis (Figure 1A and 1B) [16]. The hT/LT cells are immortalized as they have undergone >100 doublings and the hT/LT/Ras cells are transformed as indicated by their capacity for anchorage-independent growth in soft agar and athymic mice [16]. We found that the hT/LT and hT/LT/Ras cells proliferate at a similar rate which is approximately 1.5x that of primary NHBE cells (with growth factor stimulation) (Figure 1C). We also examined the size of each cell type and found that the immortalized and *ras*-transformed bronchial epithelial cells were relatively smaller than the primary epithelial cells (NHBE: 1099 ± 82 μm^3^; hT/LT: 433 ± 43 μm^3^; hT/LT/ras: 711 ± 56 μm^3^). We examined the relative glucose consumption and lactate secretion by these three cell types over 72 hours and found that both the normal and the H-Ras^V12^-transformed bronchial epithelial cells consumed more glucose and secreted more lactate than the immortalized cells during this time period as has been previously reported (Figure 1D and 1E) (glucose consumed: hT/LT *vs*. NHBE or hT/LT/Ras at 48 and 72 hours *p *< 0.05 ; lactate secreted: hT/LT *vs*. NHBE or hT/LT/Ras at 24, 48 and 72 hours, *p *< 0.005) [15]. These results confirm that H-Ras^V12 ^expression increases lactate secretion in immortalized cells but indicate that high lactate secretion may itself not be a distinct characteristic of neoplastic transformation since the primary cells also secrete increased lactate. The high production of lactate relative to glucose consumption in the primary, immortalized and transformed epithelial cells indicates that lactate is being produced from additional carbon sources such as pyruvate, glutamine and intracellular stores of glycogen.

### Intracellular pyrimidine and purine ribose synthesis from glucose are similar in primary, immortalized and transformed bronchial epithelial cells

**Figure 2:**
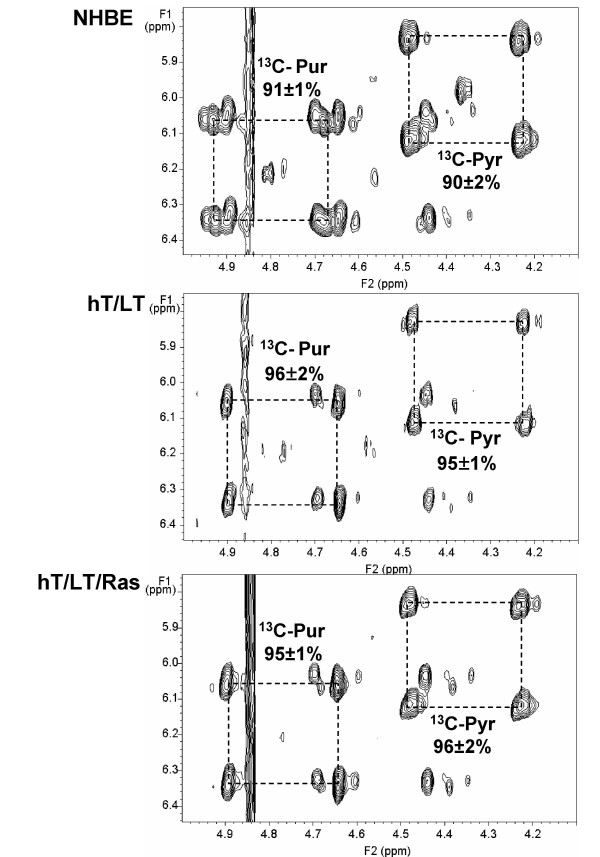
**NMR-based isotopomer analysis of intracellular pyrimidine and purine ribose synthesis from glucose**. The regions of the 2D TOCSY spectra captured from NHBE, hT/LT and hT/LT/Ras cells including both the pyrimidine and purine riboses were analyzed for relative ^13^C enrichment. The regions of the spectra showing the cross peaks of the nucleotide ribose moieties (H1'-H2' interaction) are shown.

Two-dimensional nuclear magnetic resonance spectroscopy (2D NMR) can be used to analyze the conversion of glucose carbons into soluble intracellular metabolites of anabolic pathways [17,18]. We cultivated NHBE, hT/LT and hT/LT/Ras cells in medium containing 1 gm/L [U-^13^C]-1,2,3,4,5,6-glucose and, after two population doublings, extracted the cells with 10% trichloroacetic acid and dissolved the lyophilized extract in 100% D_2_O. We minimized the time required for the cell harvest and washing to <20 minutes in order to ensure that subsequent analyses closely reflected the metabolic state present during exponential growth. We recorded the NMR spectra at 14.1 T with a spin lock field strength of 9 kHz for 50 ms, using a cold probe under standardized conditions of acquisition. ^13^C isotopomers were quantified by indirect detection of protons in the 2D total correlated spectroscopic analysis (TOCSY) NMR spectra. Figure 2 shows the region of the TOCSY spectra captured from NHBE, hT/LT and hT/LT/Ras cells that includes both the pyrimidine and purine riboses. These ^12^C-glucose-derived molecules reveal a faint single cross peak for unlabeled metabolites (center of dashed boxes), surrounded by four satellite cross peaks that reflect the ^13^C-labeled molecules (dashed box; Figure 2). The cross peak patterns near 6.2/4.8 ppm correspond to ribose in purine nucleotides (mainly ATP and ADP), and the cross peaks at 6/4.4 ppm correspond to the pyrimidine nucleotide ribose (mainly UTP and UDP). We observed only two species of the pyrimidine and purine riboses present, the first in which all carbons are ^12^C (central cross peak) and the second in which all carbons are ^13^C (outer four cross peaks; Figure 2). These two pools derived from different carbon sources and the fully labeled metabolites have been synthesized directly from the ^13^C glucose supplied in the medium. The high level of ^13^C-enrichment implies that there is considerable *de novo *nucleotide biosynthesis in all three proliferating cell populations but that the immortalized and H-Ras^V12^-transformed bronchial epithelial cells exhibited minimally increased activity compared with the untransformed cells.

### Increased conversion of glutamate/glutamine from glucose by Ras-transformed cells

**Figure 3:**
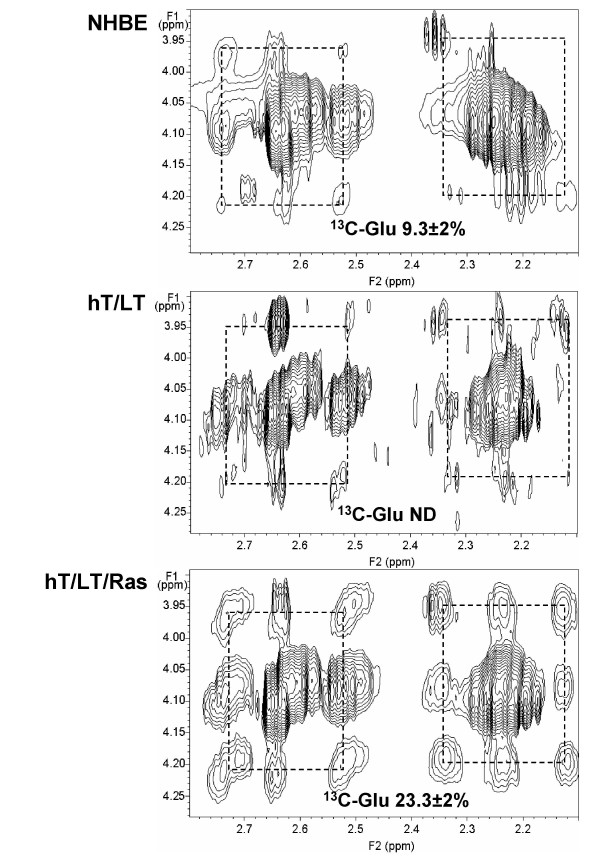
**NMR-based isotopomer analysis of intracellular glutamate/glutamine synthesis from glucose**. The regions of 2D TOCSY spectra obtained from NHBE, hT/LT and hT/LT/Ras cells specific for the combined glutamate/glutamine, were analyzed for relative ^13^C enrichment. Spectra from NHBE, hT/LT and hT/LT/Ras cells are shown. ^13^C isotopomers were quantified by indirect detection of protons in the 2D TOCSY NMR spectrum and % ^13^C-enrichment is indicated adjacent to the cross peaks.

Although glutamate and glutamine are non-essential amino acids, there is considerable *de novo *biosynthesis, as shown by the isotope incorporation. Figure 3 shows regions of the TOCSY spectra that include the C2H protons interacting with the C3H and C4H protons of free Glu, and in reduced glutathione. The pattern of the satellite peaks is more complex than for the ribose described above. In addition to the fully unlabeled molecules, there are also all possible isotopomers, corresponding to singly, doubly and triply labeled versions of the amino acids. Furthermore, the relative intensities of these isotopomers are unequal, which demonstrates the relative importance of different pathways that lead to labeled glutamate and glutamine (Figure 3). Unexpectedly, we observed a marked increase in ^13^C-enrichment of the glutamate/glutamine enrichment with H-Ras^V12^-transformation (compare hT/LT to hT/LT/Ras; ND = not detectable; Figure 3). Glutamate becomes labeled by transamination of 2-oxoglutarate produced within the mitochondria. ^13^C can enter into citrate either in 2-carbon steps by pyruvate dehydrogenase (PDH) or as three carbons via the anaplerotic carboxylation of pyruvate. These two routes produce different label pattems in 2-oxoglutarate and the specific labeling pattern indicates that both the PDH and pyruvate carboxylase entry points are active. The observation that the introduction of H-Ras^V12 ^increases their relative ^13^C-enrichment provides substantial direct evidence for the high activity of mitochondrial metabolism in H-Ras^V12^-transformed bronchial epithelial cells.

### Increased ^13^C-glucose derived aspartate and uridine in Ras-transformed cells

**Figure 4:**
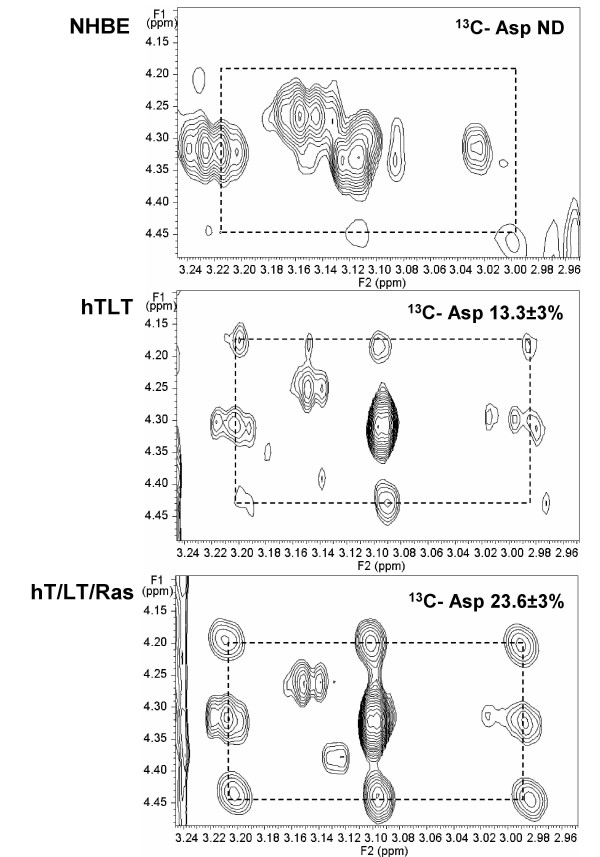
**NMR-based isotopomer analysis of intracellular aspartate synthesis from glucose**. The regions of 2D TOCSY spectra obtained from NHBE, hT/LT and hT/LT/Ras cells specific for aspartate were analyzed for relative ^13^C enrichment. Spectra from NHBE, hT/LT and hT/LT/Ras cells are shown. ^13^C isotopomers were quantified by indirect detection of protons in the 2D TOCSY NMR spectrum and % ^13^C-enrichment is indicated adjacent to the cross peaks.

**Figure 5:**
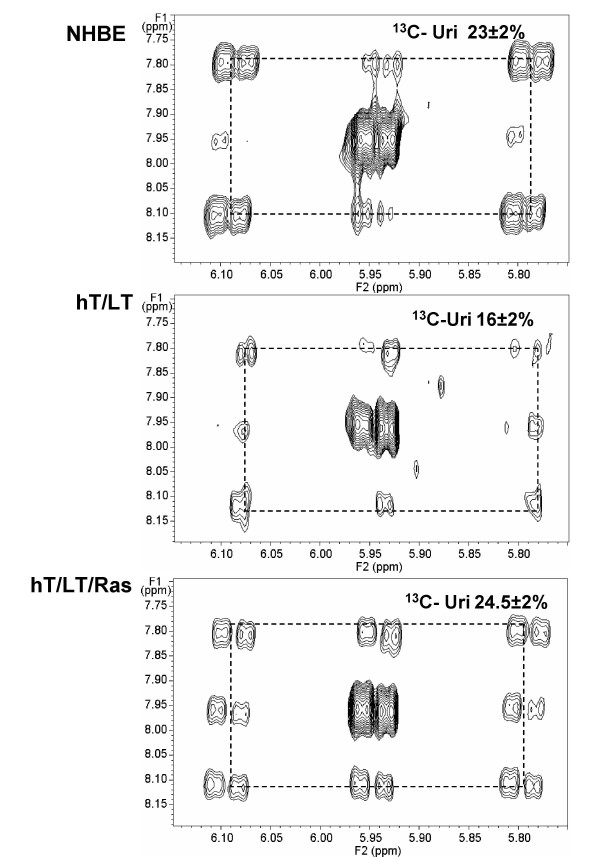
**NMR-based isotopomer analysis of intracellular uridine synthesis from glucose**. The region of the spectra showing the cross peaks of the uridine bases (H6-H5) of NHBE, hT/LT and hT/LT/Ras cells are shown. ^13^C isotopomers were quantified by indirect detection of protons in the 2D TOCSY NMR spectrum and % ^13^C-enrichment is indicated adjacent to the cross peaks.

TCA cycle intermediates may be used for biosynthesis in order to satisfy the higher demand for nucleotides in dividing cells. In Figure 4, the cross peak centered at 4.3/3.1 ppm corresponds to the unlabeled aspartate which is surrounded by the cross peaks of ^13^C-labeled aspartate (dashed boxes). We observed a stepwise increase in aspartate ^13^C-enrichment with sequential immortalization and transformation of the normal human bronchial epithelial cells (compare NHBE to hT/LT to hT/LT/Ras; Figure 4). Like glutamate, aspartate is a product of an intermediate in the tricarboxylic acid cycle (via the transamination of oxaloacetate) and these data also indicate that introduction of activated H-Ras^V12 ^increases the activity of the tricarboxylic acid cycle. Furthermore, labeled aspartate enters pyrimidine nucleotide biosynthesis. In Figure 5, the cross peak pattern centered at 8/5.95 ppm corresponds to the H6,H5 ring protons of uridine (UTP and UDP). The satellite pattern shows a mixture of isotopomers, including unlabeled, two singly labeled and a doubly labeled variant (Figure 5). The C6 and C5 of uridine derive from the α and β carbons of aspartate, respectively, which is obtained by transamination of OAA. Although most steps in pyrimidine biosynthesis are cytoplasmic, one step, catalyzed by orotate dehydrogenase, occurs within the mitochondrial matrix, and requires the availability of mitochondrial NAD+. The two singly labeled Asp and uridine rings most probably arise from the incorporation of label via PDH activity, followed by scrambling at the succinate step. These ^13^C enrichment data strongly indicate that the TCA cycle is fully active in these cells.

### hT/LT/Ras-transformed cells consume high oxygen and are especially sensitive to anoxia

**Figure 6:**
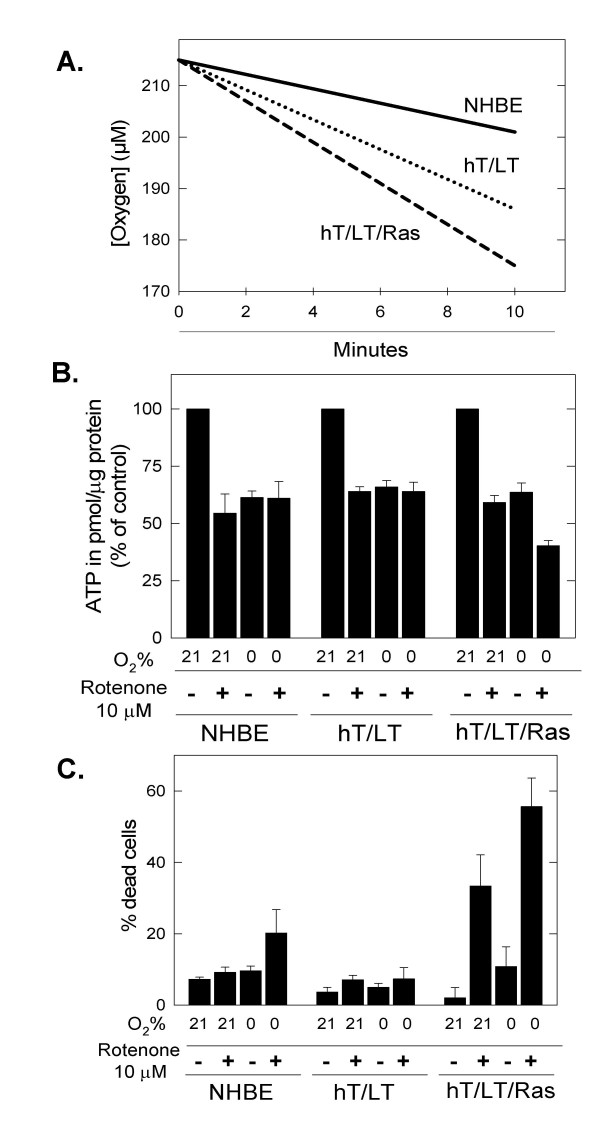
**Ras-transformed cells demonstrate increased dependence on mitochondrial respiration**. a. Oxygen consumption was measured using 5 × 10^6 ^primary (NHBE), immortalized (hT/LT) and Ras-transformed (hT/LT/Ras) bronchial epithelial cells in normoxia (21% oxygen) with a Clark type polarographic electrode. b. 5 × 10^5 ^primary bronchial epithelial cells (NHBE), immortalized (hT/LT) and transformed (hT/LT/Ras) cells were cultured in normoxia or anoxia +/- 10 μM rotenone for 24 h. The cells were washed X1, lysed and ATP was measured using a bioluminescent assay and values expressed as % of control. Cells in normoxia without rotenone were used as the control (ATP amounts of controls: NHBE 0.707 ± 0.018, hT/LT 0.734 ± 0.07, hT/LT/Ras 0.7 ± 0.038 pmol per μg protein). c. 5 × 10^5 ^NHBE, hT/LT and hT/LT/Ras cells were cultured in normoxia or anoxia with or without rotenone for 24 hrs. Viable and non-viable cells were enumerated by Trypan blue exclusion using a hemacytometer.

Based on the relative increased flux of glucose into the tricarboxylic acid cycle, we speculated that hT/LT/Ras-transformed bronchial epithelial cells may be more reliant on electron transport than primary or immortalized bronchial epithelial cells. We measured basal oxygen consumption and found that the introduction of Ras^V12^ caused an increase in oxygen consumption relative to the immortalized cells (Figure 6A). We then exposed the three cell types to atmospheric oxygen or 0% oxygen in the presence and absence of the complex I inhibitor, rotenone, and, after 24 hours, measured intracellular ATP and cell death. We found that the steady-state intracellular concentration of ATP was reduced by rotenone to a greater extent in the H-Ras^V12^-transformed cells than in the primary and immortalized cells (hT/LT/Ras *vs*. either NHBE or hT/LT, *p *< 0.05) but that anoxia similarly affected the three cell types (Figure 6B). The increased depletion of ATP by rotenone (in normoxic or anoxic conditions) was mirrored by increased cell death in the Ras^V12^-transformed cells (Figure 6C). Taken together, these data suggest that activation of Ras signaling may cause an increased reliance on the electron transport chain, a process that is tightly coupled to the observed high tricarboxylic acid cycle activity through the oxidation of NADH.

## Discussion

The high enrichment of ^13^C-glucose-derived carbons into glutamate/glutamine, aspartate and uridine in the H-Ras^V12^-transformed bronchial epithelial cells provides unambiguous evidence that the tricarboxylic acid cycle is highly active in these cells. That we observed increased pooling of the ^13^C-glucose-derived products from the tricarboxylic acid cycle in the hT/LT/Ras-transformed bronchial epithelial cells suggests either that H-Ras^V12 ^causes increased synthesis or decreased utilization of these anabolic precursors. The NHBE, hT/LT and hT/LT/Ras cells were allowed to double twice prior to extraction and NMR analysis, and we thus anticipate that the relative anabolic utilization of these precursors is not decreased by H-Ras^V12^. Coupled to the observations that oxygen consumption is increased by H-Ras^V12 ^and that the H-Ras^V12^-transformed cells are especially sensitive to electron transport perturbation by rotenone, the observed increase in ^13^C-enrichment of the glutamate/glutamine, aspartate and uridine supports an increased activity of the tricarboxylic acid cycle rather than decreased utilization of the detected anabolic substrates.

The increased ^13^C-enrichment of glutamate/glutamine and aspartate from glucose indicate increased TCA activity. The high glycolytic flux in these cells requires a means of regenerating cytoplasmic NAD+. The fermentation of pyruvate to lactate, followed by secretion of lactate into the medium, can sustain only that part of glycolytic flux that results in lactate production. Any additional glycolysis that produces pyruvate that enters the TCA (for example) needs a second means of regenerating NAD+. This may stem from the activity of the aspartate/malate NADH shuttle which previously has been found to be active in neoplastic cells [19]. Mitochondrial and cytoplasmic aspartate aminotransferases produce aspartate and glutamate from oxaloacetate and α-ketoglutarate respectively and are activated early in carcinogen-induced transformation *in vivo *[20]. Furthermore, the concentration of aspartate and glutamate are increased in human colon and gastric adenocarcinomas relative to matched normal tissues [21]. The aspartate/malate shuttle functions to transfer electrons via NADH produced from glycolysis into the mitochondria for electron transport. Given that activated H-Ras^V12 ^increases oxygen consumption and confers sensitivity to electron transport perturbation, we speculate that flux through this NADH shuttle may be increased by oncogenic Ras and future studies will address this hypothesis.

Several genetic alterations of cancer cause induction of cellular proliferation through the epidermal growth factor receptor (EGFR)-Ras-Raf-mitogen activated protein kinase/extracellular signal-regulated kinase (MAPK/ERK; MEK)-ERK-MAPK pathway, including EGFR amplifications (colorectal, pancreatic and lung cancers) and mutations (lung adenocarcinomas and glioblastoma), and activating mutations of Ras (pancreatic, papillary thyroid, colon and lung cancers) and Raf (melanoma, papillary thyroid and colon cancers) [11]. In this study, we have found that activation of this signaling cascade through ectopic expression of H-Ras^V12 ^in hT/LT-immortalized bronchial epithelial cells causes an increase in the enrichment of ^13^C-carbons from glucose into key anabolic precursors produced from tricarboxylic acid cycle intermediates. Based on these observations, we predict that downstream signaling effectors of the EGFR-Ras-Raf-MEK-ERK-MAPK pathway cause activation of key rate-limiting anabolic enzymes required for increased production of these precursors. Furthermore, we postulate that the metabolic rationale for increased pooling of these precursors (*i.e*. glutamate and aspartate) in H-Ras^V12^*-*transformed proliferating cells may be related to the need for a ready supply of substrates for the production of amino acids since these precursors are required for the *de novo *synthesis of several amino acids (*i.e*. glutamine, proline, arginine, asparagine, methionine, lysine and threonine). In support of this hypothesis, recent studies have demonstrated that Ras/ERK signaling promotes translation initiation by facilitating assembly of the preinitiation complex [22]. Accordingly, increasing the availability of anabolic substrates for the translational machinery may prove to be a key metabolic event activated by oncogenic Ras.

Recent studies by Chen *et al*. have found that metastatic breast cancer cells utilize aerobic glycolysis, coupled with the tricarboxylic acid cycle and oxidative phosphorylation to generate ATP needed for cellular proliferation [23]. They conducted proteomic analyses of breast cancer cells isolated from a stage IV breast cancer patient before and after metastatic spread to the brain in athymic mice and observed a marked increase in the expression of proteins involved not only in glycolysis but also in the tricarboxylic acid cycle, including aconitate hydratase, isocitrate dehydrogenase and mitochondrial malate dehydrogenase, and in oxidative phosphorylation, including cytochrome c oxidase subunits, NADH-ubiquinone oxidoreductases and ATP synthase chains. Ras is over-expressed in the majority of breast adenocarcinomas examined [24-26] and the observed high protein expression of tricarboxylic acid cycle enzymes provides further support for the conclusion that the activities of the tricarboxylic acid cycle and electron transport chain are increased as a result of H-Ras^V12^-transformation.

## Conclusion

Pharmacologic disruption of glycolysis is currently under development as an anti-neoplastic strategy due to the observations that tumor cells metabolize glucose rapidly and are especially sensitive to glucose deprivation [6]. 3-Bromopyruvate and 2-deoxyglucose are two compounds that inhibit the first irreversible enzyme of glycolysis, hexokinase, and suppress tumor growth *in vivo *[27-29]. In this study, we provide evidence for increased tricarboxylic acid cycle activity, oxygen consumption and energetic reliance on electron transport in H-Ras^V12^-transformed cells relative to matched, normal and immortalized cells. Based on these observations, we conclude that inhibition of mitochondrial metabolism may cause selective anti-neoplastic effects. It is noteworthy that several known cytotoxic agents function by targeting the mitochondria, including arsenite, lonidamine and betulinic acid [30]. In future studies, we will determine the precise downstream Ras effector enzymes that regulate this metabolic shift in order to develop highly targeted anti-metabolites as chemotherapeutic agents.

## Methods

### Cell lines and cell culture

Normal human bronchial epithelial (NHBE) cells were obtained from Cambrex (Walkersville, MD) and NHBE cells expressing telomerase and SV40 large T antigen (hT/LT) and activated Ras (hT/LT/Ras) were gifts from Dr. B. J. Rollins, Dana Farber Cancer Institute. All experiments with NHBE cells were conducted between 4 and 6 passages. NHBE, hT/LT and hT/LT/Ras cells were grown in media containing 1 gm/L (5.5 mM) glucose and formulated with bovine pituitary extract, recombinant human epidermal growth factor, hydrocortisone, insulin, epinephrine, tri-iodothyronine, transferrin, gentamicin, amphotericin B and retinoic acid (BEGM with SingleQuots, Cambrex, Walkersville, MD). All cells were maintained at 37°C in 5% CO_2_. For some experiments, NHBE, hT/LT and hT/LT/Ras cells were grown in 0% oxygen (and 5% CO_2_) in a modular incubator chamber at 37°C (Billups-Rothenberg, Del Mar, CA). For certain other experiments, NHBE, hT/LT and hT/LT/Ras cells were treated with 10 μM rotenone (Sigma, St. Louis, MO) for 24 hours. The cells were enumerated by direct visualization using light microscopy. Sizes of NHBE, hT/LT and hT/LT/Ras cells were determined using Imagestream and IDEAS software (both from Amnis, Seattle, WA).

For NMR experiments, after cells reached 25% confluence, the media was changed to glucose-free Dulbecco's modified Eagle medium (DMEM, Invitrogen, Grand Island, NY) supplemented with SingleQuots (Cambrex) as above and also with ^13^C labeled glucose (5.5 mM) from a sterile 20% stock solution of [U-^13^C_6_]-glucose (98% ^13^C) (Cambridge Isotopes Laboratories, Andover, MA) in phosphate-buffered saline.

### Protein extraction and Western blotting

Cells were treated with 0.25% trypsin-EDTA, washed in PBS, and lysed in 2x RIPA buffer. Protein samples were resolved on a 4–20% gradient SDS-PAGE gel and transferred to a PVDF membrane. Membranes were blocked in TBS-Tween 20 (1%) containing 5% milk. Either rabbit anti-hTERT (1:250, Rockland Immunochemicals, Gilbertsville, PA), mouse anti-β-actin (1:5000, Sigma, St. Louis, MO), rabbit anti-H-Ras (1:1000, Santa Cruz Biotechnology, Santa Cruz, CA) or mouse anti-SV40 large T-antigen antibody (1:1000, Oncogene/Calbiochem, San Diego, CA) were re-suspended in 10 ml of TBS-Tween 20 (5% milk) and incubated with the membrane for 1 hour. Secondary antibodies were goat anti-rabbit or anti-mouse HRP conjugated (1:8000, Pierce Biotechnology, Rockford, IL). All Western blotting experiments were repeated for a total of 4 experiments.

Scanned images were quantified by densitometric analyses using Quantiscan software Version 3.0 (Biosoft, United Kingdom). Values obtained were normalized to α-tubulin) (as a control) and expressed in densitometric units as a percentage of the control. All values represent the mean ± SD of 4 independent experiments.

### Lactate and glucose measurements

Lactate concentrations in the media were measured using a lactate oxidase-based assay read at 540 nm (Trinity, Wicklow, Ireland). Glucose concentrations were measured using a hexokinase-glucose-6-phosphate dehydrogenase enzymatic assay read at 340 nm (Sigma, St. Louis, MO). All data are expressed as the mean ± SD of five experiments. Statistical significance was assessed by the unpaired two-tail *t-*test.

### Metabolite extraction

Cells grown in the presence of ^13^C-labeled glucose were harvested after two population doublings by trypsinization with 0.25% trypsin-EDTA for 60 seconds at 37°C in 5% CO_2 _and low speed centrifugation. The cells were counted by direct visualization using light microscopy and equal numbers of cells were spun down, washed twice with ice-cold PBS followed by low speed centrifugation (1000 rpm) at 4°C to remove adhering medium, and then flash frozen in liquid N_2_. The cold pellet was extracted with 10% ice-cold TCA (twice), followed by lyophilization as previously described [17,18]. Dry cell extract was redissolved in 0.35 ml D_2_O with 142 μM DSS (2, 2-dimethyl-2-silapentane-5-sulfonate sodium salt) as both a chemical shift reference and as a concentration standard and loaded into a 5 mm Shigemi tube.

### Nuclear Magnetic Resonance

All NMR spectra were recorded at 14.1 T on Varian Inova NMR spectrometer at 20°C using a 90° excitation pulse. 1D spectra were recorded using an acquisition time of 2 seconds and a relaxation delay of 3 seconds during with the residual HOD signal was suppressed using a weak transmitter pulse (ca. 20 Hz B_1 _field). For analyzing the cellular extracts and determining the positional enrichment with ^13^C we used 2D experiments (TOCSY and HSQC), and analyzing the satellite peaks in the TOCSY as described in detail [31,32]. TOCSY experiments were recorded with a 6000 Hz spectral width both dimensions, 0.341 s acquisition time in t_2 _and 0.05 s in t_1_, a recycle time of 2 s, a 50 ms isotropic mixing time, and a B_1 _field strength of 8 kHz generated with MLEV-17. The data tables were zero filled to 8192 by 2048 complex points, apodized using an unshifted Gaussian function and 0.5 Hz line broadening exponential in both dimensions prior to double fourier transformation.

Metabolites were assigned based on their ^1^H and ^13^C chemical shifts, and TOCSY connectivity patterns[17,33,34], and compared with our in-house data base of standards recorded under identical conditions [31]. Metabolite concentrations were determined by comparing the area of assigned resonances to that of the DSS methyl group (9 protons) according to Eq (1):

c(S) = c(DSS).A(s)n(S)/9A(DSS)

Where c(S) is the concentration of the desired solute, c(DSS) is the concentration of the DSS standard, A(s), A(DSS) are the areas of the solution and DSS resonances, and n is the number of protons in the solute peak (e.g. 3 for lactate methyl protons). With the recycle time used, the degree of saturation was small under the conditions of the experiments. Saturation factors were assessed by independent measurements of the T_1_values using inversion recovery, according to:

M(obs) = M(true) (1-exp-R_1_D)

Where M(obs), M(true) are the observed and fully relaxed magnetizations, R_1 _is the spin-lattice relaxation rate constant and D is the recycle time.

### Isotope enrichment from NMR

^13^C enrichment was determined from peak areas in 1D spectra for well-resolved metabolites such as the lactate methyl group. Peak areas of the satellite, A(^13^C), and central, A(^12^C), resonances were determined by integration after careful phasing and baseline flattening. The %^13^C was then calculated according to Eq. (3):

F = 100A(^13^C)/[(A(^13^C)+A(^12^C)]

For metabolites poorly resolved in 1D spectra, we used the 2D TOCSY method as previously described [31,32]. The base-planes were carefully corrected, and cross peak volumes were determined by volume integrated using VNMR. This approach has been shown to provide very accurate, and unbiased estimates of the ^13^C content. The various isotopomer enrichments were calculated by replacing the peak area with peak volumes in equation 3. In these TOCSY experiments, the protons were partially saturated owing to the shorter recycle time (2 s). Thus, the actual peak volumes were corrected according to the differential T_1 _values of protons attached to ^13^C or ^12^C according to Eq. (2). Effective T_1 _values were determined on standards recorded under the same solvent conditions using the inversion recovery sequence.

Analytical precision was determined by replicate analysis of symmetry related cross peaks, and on spectra transformed using slightly different window functions. The errors were then estimated with respect to the signal to noise ratios of the peaks. The number of biological replicates was 3.

### Oxygen consumption

NHBE, hTLT and hT/LT/Ras cells in culture were detached, washed × 1 with PBS and resuspended in complete BEGM medium at 10^7^cells/ml. Oxygen consumption was measured using 5 × 10^6 ^cells in 500 μL medium at 37°C using a Clark-type polarographic electrode (Strathkelvin Mitocell MT 200, Motherwell, United Kingdom). A starting O_2 _concentration of 215 μM was assumed based on O_2 _solubility at sea level at 37°C [35-37]. Experiments were repeated for a total of five times. The data shown are the results of a single representative experiment.

### ATP measurements

5 × 10^5 ^NHBE, hTLT and hT/LT/Ras cells in culture for 24 hrs in normoxia (21% oxygen) or anoxia (0% oxygen) +/- 10 μM rotenone were washed (while still adherent) with cold PBS ×1. Passive lysis buffer (1X, Molecular Probes, Invitrogen, Carlsbad, CA) was then added directly to the plates and the cells immediately harvested by scraping. The lysates were flash frozen (to -80°C) and thawed (to 37°C) once to accomplish complete lysis and then centrifuged (at 4°C) for 30 seconds to clear the lysates. Intracellular ATP levels were determined using a bioluminescence assay (Molecular Probes) utilizing recombinant firefly luciferase and its substrate, D-luciferin and following manufacturer's instructions. The luminescence was read in a TD-20/20 luminometer (Turner Designs, Sunnyvale, CA) at 560 nm. The ATP values were calculated using an ATP standard curve. The protein concentrations of the lysates were estimated using the bicinchoninic acid (BCA) assay (Pierce Biotechnology, Rockford, IL) and ATP was expressed as pmol per μg protein. All data are expressed as the mean ± s.d. of five experiments. Statistical significance was assessed by the unpaired two-tail *t-*test.

### Cell viability

5 × 10^5 ^NHBE, hTLT and hT/LT/Ras cells were plated in normoxia or anoxia with or without 10 μM rotenone for 24 hrs, detached (with 0.25% Trypsin/EDTA) and counted using a hemacytometer. Cell viability was assessed by Trypan blue exclusion. Trypan blue solution was prepared by combining 0.5 ml of Trypan blue (Sigma, St Louis, MO) with 0.3 ml of PBS. Cells in suspension were mixed 1:1 with Trypan blue solution and incubated at room temperature for 5 min. The numbers of unstained (viable), stained (dead) and total cells were counted in a hemacytometer by two independent observers and the percentage of viable cells calculated. The numbers of cells counted in the hemacytometer were between 100–500 (1–5 × 10^6 ^cells/microliter). All data are expressed as the mean ± s.d. of five experiments. Statistical significance was assessed by the unpaired two-tail *t-*test.

## Abbreviations

TCA: tricarboxylic acid cycle;

GDH: glutamate dehydrogenase;

GS: glutamine synthetase;

2OG: 2-oxoglutarate;

HSQC: heteronuclear single quantum coherence;

OAA: oxalacetate;

TAg SV40 large: T Antigen;

TOCSY: Total Correlation Spectroscopy.

## Competing interests

The author(s) declare that they have no competing interests.

## Authors' contributions

ST conducted all NMR and rotenone sensitivity experiments and participated in the conception and drafting of the manuscript.

ANL interpreted the NMR spectra data.

KKN conducted the oxygen consumption experiments.

SA captured and processed the NMR spectra.

JC conceived the research, directed all experiments and drafted the manuscript.

All the authors have been involved in the drafting of the manuscript and have read and approved the final manuscript.
